# Anti-Obesity Effects of Macroalgae

**DOI:** 10.3390/nu12082378

**Published:** 2020-08-08

**Authors:** Saioa Gómez-Zorita, Maitane González-Arceo, Jenifer Trepiana, Itziar Eseberri, Alfredo Fernández-Quintela, Iñaki Milton-Laskibar, Leixuri Aguirre, Marcela González, María P. Portillo

**Affiliations:** 1 Nutrition and Obesity Group, Department of Pharmacy and Food Science, Faculty of Pharmacy, University of the Basque Country (UPV/EHU) and Lucio Lascaray Research Institute, 01006 Vitoria, Spain; saioa.gomez@ehu.eus (S.G.-Z.); maitanega14@gmail.com (M.G.-A.); jenifer.trepiana@ehu.eus (J.T.); itziar.eseberri@ehu.eus (I.E.); alfredo.fernandez@ehu.eus (A.F.-Q.); mariapuy.portillo@ehu.eus (M.P.P.); 2 CIBEROBN Physiopathology of Obesity and Nutrition, Institute of Health Carlos III, 01006 Vitoria, Spain; 3 Bioaraba Health Research Institute, 01006 Vitoria, Spain; 4 Nutrition and Food Science Department, Faculty of Biochemistry and Biological Sciences, National University of Litoral and National Scientific and Technical Research Council (CONICET), Santa Fe 3000, Argentina; maidagon@fbcb.unl.edu.ar

**Keywords:** macroalgae, seaweed, obesity, triglyceride, adipose tissue, adipocyte

## Abstract

Macroalgae have attracted great interest for their potential applications in nutraceutical and pharmaceutical industries as source of bioactive medicinal products and food ingredients. This review gathers data from *in vitro* and *in vivo* studies addressing the anti-obesity effects of macroalgae. Great consensus exists in all reported *in vitro* studies concerning the reduction induced by seaweed extracts in the expression of transcriptional factors controlling adipogenesis. In animals, macroalgae reduced body fat accumulation and prevented other obesity features, such as dyslipidemia, insulin resistance and fatty liver. These effects are not due to food intake reduction, since few studies have reported such event. Indeed, the effects on metabolic pathways in target tissues/organs seem to play a more relevant role. Macroalgae can reduce de novo lipogenesis, limiting fatty acid availability for triglyceride synthesis in white adipose tissue. This effect has been observed in both cell cultures and adipose tissue from animals treated with macroalgae extracts. In addition, increased fatty acid oxidation and thermogenic capacity, as well as a shift towards healthier gut microbiota composition may contribute to the body fat-lowering effect of macroalgae. Studies in humans are needed to determine whether macroalgae can represent a feasible tool to prevent and/or manage overweight and obesity.

## 1. Introduction

For several centuries, seaweeds, also known as macroalgae, have been an important dietary component in Asian countries such as China, Japan and Korea, where they have also been used in alternative medicine. In Western and European countries, their use has become more popular over the past few decades [1,2], being used to improve nutritional, textural, organoleptic and sensorial properties of food products [3]. Macroalgae are taxonomically classified within three main groups: *Chlorophyceae* (green algae), *Rhodophyceae* (red algae) and *Phaeophyceae* (brown algae).

Seaweeds represent a good source of both micro- and macronutrients. In fact, it is well known that they are rich in minerals, particularly iron and magnesium, as well as in water-and lipid-soluble vitamins [1]. With regard to macronutrients, marine algae are a good source of proteins, containing all the essential amino acids. In addition, they are also of interest due to their richness in sulfated polysaccharides, which vary according to phyla; the main sulfated polysaccharides in marine algae include ulvan (green algae), carrageenan (red algae), fucoidan and laminarans (brown algae) [4]. Another portion of carbohydrates present in macroalgae belongs to dietary fiber, which promotes the improvement of digestive tract health [5]. Moreover, current evidence points that macroalgae also contain phytochemicals, including polyphenols and carotenoids, with potential prebiotic activity [5]. Despite their low lipid content, macroalgae contain substantial amounts of polyunsaturated fatty acids (PUFAs), such as long-chain ω-3 polyunsaturated fatty acids (docosahexaenoic acid, DHA, and eicosapentaenoic acid, EPA). Indeed, it is important to point out that they are considered as a rich source of those nutrients compared to other animal and plant-derived foods [5].

There is evidence showing that macroalgae and their extracts show anti-oxidant, anti-microbial, anti-inflammatory, anti-cancer, anti-diabetic, anti-hypertensive, anti-hyperlipidemic and anti-obesity effects [3]. Taking into account that obesity has become a major health problem due to the fact that its prevalence has increased substantially in the last decades and also because it is a major risk factor for a range of chronic diseases including diabetes, cardiovascular diseases and cancer [6,7,8,9], the present review focuses on the anti-obesity effects of macroalgae. It is important to emphasize that nowadays few drugs are available for obesity treatment [10]. This scenario highlights the necessity to look for new agents with anti-obesity activity, especially from biological sources.

Although several bioactive compounds, such as phlorotannins, fucoxanthin, fucoidans and alginates, have been reported as responsible for the anti-obesity effects of algae, the present review gathers data from *in vitro* and *in vivo* studies in which algae or algae extracts were used, but not those using isolated algae compounds. The schema followed to present the reported information is to describe the results obtained and then to summarize the mechanisms explaining these results, in each study

## 2. *In Vitro* Studies

**Figure 1:**
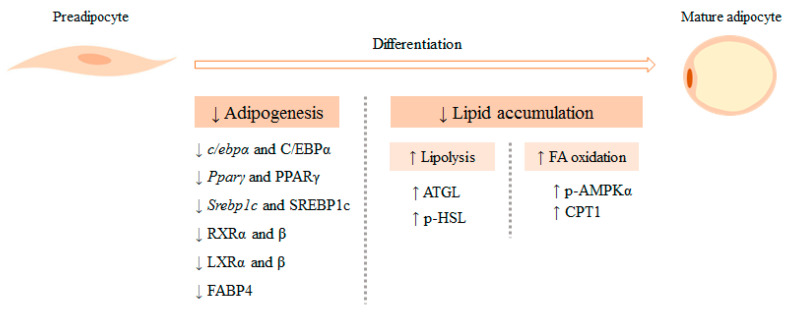
Anti-obesity mechanisms of action described in *in vitro* studies. ATGL: adipose triglyceride lipase, C/EBPα: CCAAT-enhancer-binding protein alpha, CPT1: carnitine palmitoyltransferase I, FABP4: fatty acid-binding protein 4, HSL: hormone sensitive lipase, LXRα: liver X receptor α, LXRβ: liver X receptor β, p-AMPKα: phophorilated-AMP-activated protein kinase alpha, PPARγ: peroxisome proliferator activated receptor γ, RXRα: retinoid X receptor α; RXRβ: retinoid X receptor β; SREBP1c: sterol regulatory element-binding protein 1-c, ↓: decreased, ↑: increased.

To date, several *in vitro* studies have been conducted in pre-adipocytes and mature adipocytes from the 3T3-L1 line, one of the most commonly used cell lines [11], to analyze the effects of algae extracts on adipogenesis and metabolic processes involved in triglyceride (TG) accumulation (Table 1; Figure 1).

In this line, Kim et al. [12] studied whether an extract of the brown macroalgae *Sargassum thunbergii*, also named gulf-weed or sea holly and known to contain terpenoids, polysaccharides and polyphenols, was able to modulate adipogenesis. Bearing this in mind, murine 3T3-L1 pre-adipocytes were treated with 10, 50 or 100 µg/mL of the extract during the 8 days of the differentiation period. All the tested doses of the macroalgae extract effectively reduced lipid content in a dose-dependent manner, indicating adipogenesis inhibition. To elucidate the mechanisms underlying this effect, the expression of several transcription factors involved in adipogenesis were studied. The treatment with *Sargassum thunbergii* extract significantly decreased mRNA and protein levels of peroxisome proliferator activated receptor γ (PPARγ), sterol regulatory element-binding protein 1 c (SREBP1c) and CCAAT-enhancer-binding protein α (C/EBPα) at all doses tested. In order to gain more insight, the expression of proteins that heterodimerise with PPARγ to promote target gene expressions were also measured. In this regard, the protein expressions of retinoid X receptor α and β (RXRα and RXRβ, respectively), as well as those of liver X receptor α and β (LXRα and LXRβ, respectively) were reduced by the extract in a dose-dependent manner. All these results suggest that *Sargassum thunbergii* exerts anti-adipogenic effects by modulating the PPARγ pathway.

In another study, Kang et al. [13] tested the anti-adipogenic effect of ethanol extracts from brown, green and red macroalgae, collected in the coast of Jeju Island (South Korea), in 3T3-L1 pre-adipocytes. For this purpose, cells were treated during the differentiation period with 100 µg/mL of brown, green or red macroalgae extracts (14, 5 and 8 algae extracts, respectively), and the lipid content of cells was determined by Oil red O staining. Among the 27 tested extracts, 13 were effective in reducing the lipid content, being the extract of the red macroalgae *Plocamium telfairiae* the most effective one. Based on these observations, in an additional experiment, 3T3-L1 pre-adipocytes were treated with 25, 50 or100 µg/mL of *Plocamium telfairiae*, and the authors observed that all doses reduced the cell lipid content. In order to elucidate the underlying mechanisms, the protein expressions of PPARγ, C/EBPα, SREBP1-c and fatty acid-binding protein 4 (FABP4) were measured. It was observed that the expression of the three transcription factors was reduced after cell treatment with the three doses. Thus, they concluded that some extracts from brown, red and green macroalgae could be effective in reducing lipid content of maturing pre-adipocytes, and that *Plocamium telfairiae* had a potent anti-adipogenic effect.

The same research group conducted another study [14] aimed to analyze the effect on adipogenesis of an extract from the edible red macroalgae *Gelidium amansii* (GAE), an algae included in the diet long ago due to its anti-oxidant, anti-carcinogenic and immunomodulatory effects. For this purpose, post-confluent 3T3-L1 pre-adipocytes were induced to differentiation (8 days) and treated with 50 or 100 µg/mL of GAE during the adipogenic process. Cells treated with both extract doses expressed lower protein levels of PPARγ and SREBP1c, whereas C/EBPα was only reduced in cells treated with the highest dose. These results denote that *Gelidium amansii* has an anti-adipogenic effect in 3T3-L1 pre-adipocytes.

Sharma et al. [15] analyzed whether an extract obtained from the green macroalgae *Caulerpa okamurae*, a seaweed rich in minerals, fiber, vitamin A, vitamin C, alkaloids, β-sitosterol and essential unsaturated fatty acids, could exert beneficial effects on obesity. For this purpose, both *in vitro* and *in vivo* studies were carried out. In the *in vitro* study, 3T3-L1 pre-adipocytes were differentiated (8 days), adding the algae extract (25, 50, 125, 250 or 500 µg/mL) to the medium. At the end of the experiment, none of the tested extracts showed cytotoxicity. As far as lipid accumulation is concerned, all tested doses significantly decreased TG content when compared with the control cells. Among the treated pre-adipocytes, those receiving the extract at 250 or 500 µg/mL doses showed the greatest TG reductions. In order to analyze the protein expression of the main transcription factors regulating adipogenesis, the 50 and 250 µg/mL doses were selected. In this regard, both doses effectively decreased the expression of PPARγ and C/EBPα, in comparison to the non-treated cells. By contrast, the protein expression of SREBP1-c was only lowered in the cells treated with the 250 µg/mL dose. Based on these results, the authors concluded that *Caulerpa okamurae* could represent a promising natural agent in the prevention of obesity.

Later on, Seo et al. [16] demonstrated the anti-obesity properties of an extract obtained from the brown macroalgae *Ishige okamurae*. In this study, murine 3T3-L1 pre-adipocytes, treated with 6.25, 12.5 or 25 µg/mL of the extract during the differentiation period (8 days), showed dose-dependent inhibition of adipogenesis. Indeed, a reduction in lipid accumulation in cells was detected due, at least in part, to a reduction in the protein expression of the adipogenic transcription factors C/EBPα and PPARγ. Moreover, *Ishige okamurae* extract also increased the phosphorylation of the AMP-activated protein kinase alpha (AMPKα), a sensor of cellular energy status, in a dose-dependent manner. Further, the authors also observed a dose-dependent increase in both, the protein expression of adipose triglyceride lipase (ATGL) as well as the phosphorylation of hormone sensitive lipase (HSL) in mature 3T3-L1 adipocytes. Since phosphorylated AMPK promotes mitochondrial fatty acid uptake for energy production via β-oxidation, these facts led to the observed reduction in cell lipid accumulation. Finally, the extract also increased carnitine palmitoyltranferase 1 (CPT1) protein expression in 3T3-L1 cells but in this case only when the cells were treated with the highest dose (25 µg/mL). Consequently, the authors concluded that the extract obtained from *Ishige okamurae* reduced lipid storage in 3T3-L1 adipocytes, not only by decreasing adipogenesis but also by increasing lipolytic and oxidative pathways.

Lee et al. [17] analyzed the effects of a 60% ethanol extract of *Grateloupia elliptica* (GEE), a red seaweed from Jeju Island in Korea, on lipid accumulation and adipogenesis in 3T3-L1 adipocytes. Cells were treated during adipocyte differentiation (8 days) with 25, 50, 100 or 200 µg/mL of the seaweed extract. After having confirmed the absence of cytotoxicity, the results showed that GEE significantly reduced lipid accumulation at all tested doses. In addition, this reduction occurred in a dose-dependent manner, with reductions of 28%, 34%, 40% and 61% at the concentrations of 25, 50, 100 and 200 µg/mL, respectively. Moreover, the authors measured the protein expression of SREBP1, PPARγ and FABP4. The treatment with GEE extract reduced the expression of the three adipogenic proteins, suggesting an inhibitory effect on lipid accumulation and adipogenesis in pre-adipocytes.

As far as mature adipocytes are concerned, only one study has been reported so far addressing the effects of macroalgae extracts on TG accumulation. In such study, Martínez-Villaluenga et al. [18] analyzed the effect of two doses of an extract from the brown macroalgae *Undaria pinnatifida*. 3T3-L1 mature adipocytes were treated on day 8 after differentiation for 24 h with 1 or 10 µg/mL of the extract. Both doses significantly reduced TG content by 21% and 25%, respectively, without differences between both doses.

In summary, the studies carried out in cell cultures clearly show that different seaweeds belonging to the groups of brown, red and green macroalgae are able to reduce adipogenesis by inhibiting the expression of the transcriptional factors regulating this process. In addition, in some of the reported studies a dose-dependent response pattern was appreciated. Very often, the concentrations of bioactive compound used in cell cultures are clearly higher than those found in plasma and/or cells when provided as a supplement to animals, and this represents an important limitation of *in vitro* studies. In this regard, after a detailed read of the published studies, it can be seen that the authors have not indicated whether, taking into account the concentrations of the extracts studied, the experimental designs reflect the situation that occurs in the environment of adipocytes in adipose tissue when animals are supplemented with extracts of algae. Moreover, due to the fact that all the experiments were carried out in the murine line 3T3-L1, it is more difficult to extrapolate these results to human. For that reason, further studies in human adipocytes or in human explants are needed. This kind of studies are necessary since the response to algae extracts could be different from that observed in murine cells. Finally, to better understand how the extract affect adipogenesis, their effect on each stage of adipocyte differentiation should also be studied.

## 3. Animal Studies

**Figure 2:**
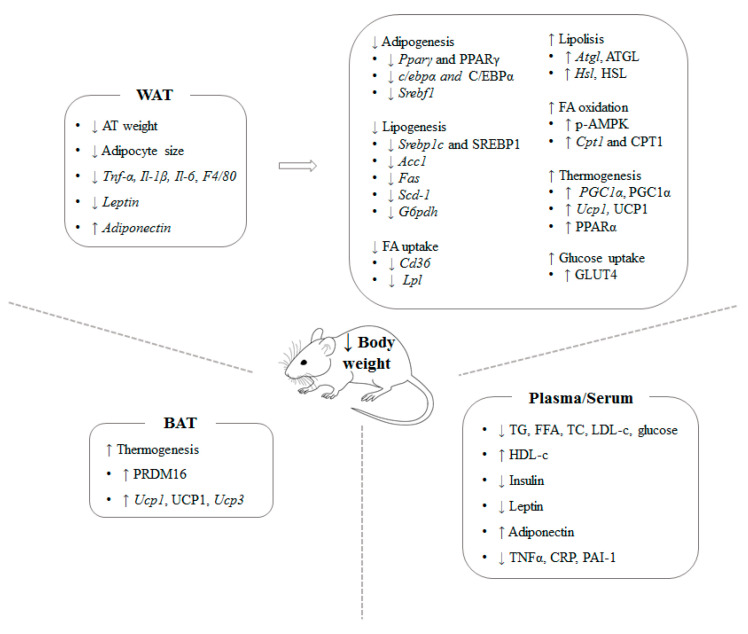
Anti-obesity effects and mechanisms of action described in *in vivo* studies. ACC: Acetyl-CoA carboxylase, AT: adipose tissue, ATGL: adipose triglyceride lipase, BAT: brown adipose tissue, Cd36: cluster of differentiation 36, C/EBPα: CCAAT-enhancer-binding protein α, CPT1: carnitine palmitoyltransferase 1, CRP: C reactive protein, F4/80: homologue in mouse to epidermal growth factor-like 1 in humans, FA: fatty acid, FAS: fatty acid synthase, FFA: free fatty acid, G6PHD: glucose 6 phosphate dehydrogenase, GLUT4: glucose transporter type 4, HDL-c: high-density lipoprotein cholesterol, HSL: hormone sensitive lipase, Il-1β: interleukin 1 β, Il-6: interleukin-6, LDL-c: low-density lipoprotein cholesterol, LPL: Lipoprotein lipase, PAI-1: plasminogen activator inhibitor-1, p-AMPK: phosphorylated AMP-activated protein kinase, PGC1α: Peroxisome proliferator-activated receptor gamma co-activator 1 alpha, PPARα: perosyxome proliferator activated receptor alpha, PPARγ: peroxisome proliferator activated receptor gamma, PRDM16: PR domain-containing 16, SCD-1: stearoyl-CoA desaturase-1, Srebf1: sterol element binding transcription factor 1, SREBP1: sterol regulatory element-binding protein 1, TC: total cholesterol, TG: triglycerides, TNF-α: tumor necrosis factor α, UCP1: uncoupling protein, UCP3: uncoupling protein 3, WAT: white adipose tissue, ↓: decreased, ↑: increased.

Numerous studies have been addressed using animal models and different experimental approaches to analyze the potential anti-obesity effect of algae. Some macroalgae revealed beneficial effects on body weight management and energy metabolism (Table 2, Table 3 and Table 4; Figure 2).

### 3.1. Brown Seaweed

Okada et al. [19] performed a study on 3-week-old male KK-Ay mice, adding lipids from *Undaria pinnatifida*, an Asian seaweed commonly known as wakame, to a standard diet (1%) or to water (0.2% by using an emulsion) during 4 weeks. At the end of the experimental period, decreased body weight was found in the animals receiving the *Undaria pinnatifida* in the water. By contrast, no change in this parameter was found in the animals receiving the seaweed in the diet. Concerning white adipose tissue, mesenteric depot was reduced in the group receiving the seaweed in the diet, while no change was observed in the animals receiving the *Undaria pinnatifida* in water. Nevertheless, the sum of the weights of different adipose tissue depots (mesenteric, epididymal, perirenal and retroperirenal) remained unchanged in both algae-treated groups when compared to the control group. In order to analyze the mechanisms underlying the effects on body and adipose tissue weights, uncoupling protein 1 (UCP1) gene and protein expressions were studied, and no changes were observed.

*Ecklonia cava* is a widely distributed brown macroalgae, rich in polyphenols. In a study carried out by Park et al. [20] the anti-obesity effect of a polyphenol-rich extract of *Ecklonia cava* was analyzed in 6-week-old male C7BL/6 mice fed either a normal diet (5% of energy from fat) or a high-fat diet (60% of energy from fat) for 3 weeks. Then, a group of high-fat diet fed mice was maintained on this feeding protocol for 8 additional weeks, while the rest of the high-fat diet fed mice were distributed in two groups, fed the high-fat diet and supplemented with extracts of Jeju *Ecklonia cava* (JEC group) or Gijang *Ecklonia cava* (GEC group) at a dose of 200 mg/kg/d. The seaweed supplementation was administered by oral intubation. The polyphenol content of the extracts was 68.8 and 79.7 mg/g for Jeju and Gijang *Ecklonia cava,* respectively, and among the phenolic compounds, the most abundant in both extracts was eckol.

Once the experimental period was completed, mice from HFD and JEC groups showed significantly higher body weights and body weight gains than mice in the GEC group, which presented a weight similar to that observed in normal diet fed mice. These changes were not due to a decrease in food intake, which was similar among all the animals fed the HFD. Consequently, the food efficiency ratio (FER; [weight gain (g)]/[total food intake (g)] × 100]) of GEC group was lower than that of the HFD group. Regarding adipose tissue, the sizes of subcutaneous, epididymal, perirenal and mesenteric depots were reduced in the GEC group to levels similar to those found in normal diet-fed mice, while not such effect was appreciated in the JEC group. These results suggest that Gijang *Ecklonia cava* was more effective than Jeju *Ecklonia cava* in obesity prevention. In plasma, mice from the HFD group had higher TG, total cholesterol, HDL-cholesterol, LDL-cholesterol and glucose levels than mice fed the normal diet. Animals in the GEC group showed reduced concentrations of total cholesterol, LDL-cholesterol and glucose compared with the HFD group.

In order to determine the potential mechanism involved in the anti-obesity effects of the tested seaweed extracts, peroxisome proliferator-activated receptor γ2 (*Pparγ2*), *C/ebpα*, *Srebp-1c* and fatty acid synthase (*Fas*) mRNA levels were measured in epididymal adipose tissue as adipogenic markers. The obesogenic diet did not modify mRNA levels of none of the genes, with the exception of *Fas* that was significantly reduced. The expression of the genes in JEC mice was similar to that of the HFD mice, while GEC mice significantly increased all of them. When the phospho-AMPK/total AMPK ratio was calculated, a reduction was found in the HFD group when compared to animals fed the normal diet.

As expected, obesity caused a low-grade chronic inflammation, which was reflected by increased mRNA levels of the pro-inflammatory cytokines tumor necrosis factor α (*Tnf-α*), interleukin 1β (*Il-1β*) and of the macrophage marker *F4/80* induced by the high-fat diet feeding. GEC mice had lower *Tnf-α*, *Il-1β*, and *F4/80* mRNA levels compared to HFD mice. In conclusion, Gijang *Ecklonia cava* improves obesity and modulates the inflammatory state of the white adipose tissue

In a similar mice model (4-week-old male C7BL/6 mice), Eo et al. [21] carried out an experiment in which the animals were fed an isocaloric diet (ND group, 10% of energy from fat) or a high-fat diet (45% of energy from fat) for 10 weeks. At this point, mice fed the obesogenic diet were divided in 3 different groups: the HFD group (fed the obesogenic diet alone), the LE group (fed the obesogenic diet supplemented with Jeju *Ecklonia cava* extract at a dose of 100 mg/kg/d) and the HE group (fed the obesogenic diet supplemented with Jeju *Ecklonia cava* extract at a dose of 500 mg/kg/d) for 12 additional weeks. The 28.2% of the extract were polyphenols, being dieckol the most abundant one.

The obesogenic diet significantly increased body weight when compared to the ND group, and only HE mice showed a reduction on this parameter when compared to the HFD group. Macroalgae supplementation did not modify the food intake compared with high-fat diet fed animals, although the HE group ate less than LE group. As expected, total adipose tissue weight, as well as the weights of visceral (epididymal, mesenteric, retroperitoneal) and subcutaneous adipose depots were higher in the high-fat diet fed animals. Total adipose tissue amount was only reduced in the group receiving the highest dose of algae extract, but without reaching the levels found in the ND group. By contrast, both seaweed extract doses reduced visceral and mesenteric adipose tissue weights. Epididymal fat depot was reduced only by the lowest dose, whereas retroperitoneal was reduced by the highest one. Finally, none of the tested doses modified the weight of subcutaneous fat depot.

In plasma, the obesogenic diet increased TG, total cholesterol and atherogenic index, whose increase was prevented by the seaweed extract. Nevertheless, no differences were observed in HDL-cholesterol among groups. As far as adipokines are concerned, leptin levels were increased in the HFD group in comparison to the ND group. This increment was avoided in mice of the LE group, whereas the decrease appreciated in the HE group remained between the levels appreciated in the ND and HFD groups. No differences regarding adiponectin concentration were reported. The leptin/adiponectin ratio, a marker of adipose tissue dysfunction that has been related to inflammation and oxidative stress, was higher in HFD mice than in ND mice, while it was normalized in HE mice.

The group of Park et al. [22] carried out another study in which the effect of a polyphenol-rich fraction of Gijang *Ecklonia cava* was studied on 6-week-old male C7BL6 mice. In this experiment, mice were fed either a normal diet (5% kcal from fat) or a high-fat diet (60% kcal from fat) for 6 weeks. Then, mice fed the obesogenic diet were fed in the same high-fat diet and supplemented or not with 300 mg/kg/day of the Gijang *Ecklonia cava* extract (EC and HFD group, respectively) for 10 weeks more. The supplementation was carried out by oral intubation.

At the end of the experimental period, the body weight increase observed in mice fed the obesogenic diet was partially prevented by the algae extract, without modifying the amount of consumed food. The lower body weight was due to a decreased fat mass (visceral and subcutaneous fat depots), without changes in lean mass. Regarding serum lipid profile, TG, total cholesterol, HDL-cholesterol and LDL-cholesterol levels were increased in mice fed the obesogenic diet while the polyphenol-rich extract partially prevented these changes. The atherogenic index, calculated as (total-cholesterol − HDL-cholesterol)/HDL-cholesterol, and related to cardiovascular disease, revealed that dyslipidemia was improved in the EC group.

These studies carried out in mice suggest that *Ecklonia cava* has potential anti-obesity effects, due to adipose tissue reduction on mice treated with this seaweed. Since this study was focused on liver steatosis, no analysis of the mechanisms underlying the decrease in fat mass was addressed.

Oh et al. [23] analyzed the effects of four brown seaweeds (*Undaria pinnafitida*, *Laminaria japonica*, *Sargassum fulvellum*, and *Hizikia fusiforme*) on body weight, adipose tissue weight and obesity-associated adipose tissue inflammation, bone-marrow-derived immune cells and systemic insulin resistance in mice showing a high-fat diet induced obesity. For that purpose, 5-week-old male C57BL/6N mice were randomly divided into six groups: LFD group (fed a diet providing 10% of energy from fat), HFD group (fed a diet providing 60% of energy from fat) and four groups fed a high-fat diet supplemented with 5% of each seaweed. The experimental period length was of 16 weeks. None of the seaweeds reverted the body weight increase induced by the high-fat diet. Surprisingly, the supplementation with *Undaria pinnafitida* induced greater body weight gain and subcutaneous adipose tissue weight, which was accompanied by a reduction in plasma adiponectin concentration. No changes were observed among experimental groups in plasma interleukin 6 (IL6) concentrations. Regarding *Laminaria japonica*, supplementation with this seaweed reduced insulin resistance in mice, as well as their blood glucose levels. Moreover, despite no change on body weight being reported, supplementation with *Sargassum fulvellum* and *Hizikia fusiforme* also reduced blood glucose. On the other hand, with the exception of *Undaria pinnafitida*, all the seaweeds reduced plasma leptin concentrations.

The authors also measured the number of dead adipocytes, adipocyte size and the presence of crown-like structures (CLS; groups of dead adipocytes) in gonadal and subcutaneous adipose tissues. The accumulation of bone-marrow-derived immune cells in adipose tissue, together with the consequent adipocyte death, is one of the complications occurring in obesity and insulin resistance. In fact, adipocyte death is related not only to bone-marrow-derived immune cells recruitment but also to CLS formation and pro-inflammatory gene expression. Supplementation with *Undaria pinnafitida* reduced the CLS number in the gonadal adipose tissue when compared to the HFD fed group. Finally, the authors isolated bone-marrow-derived immune cells from femoral and tibia bone marrow (of one representative mouse of each experimental group), which were differentiated into macrophages and stimulated or not with lipopolysaccharide (LPS) for 24 h. The authors observed that under LPS stimulation, high-fat diet fed mice derived bone-marrow-derived immune cells showed higher Il1β secretion than those of the low-fat diet or high-fat diet + seaweed fed-mice-derived. In addition, the LPS-stimulated IL6 secretion induced by high-fat diet was reverted only by *Laminaria japonica* supplementation. According to these results, the authors concluded that brown seaweeds are capable of attenuating insulin resistance by reducing adipose tissue inflammation in a mice model of dietary induced obesity. More specifically, although mice supplemented with *Laminaria japonic* remained obese, the insulin resistance induced by the high-fat diet was improved, and the release of pro-inflammatory cytokines reduced. Moreover, dietary treatment with *Undaria pinnafitida*, *Sargassum fulvellum* and *Hizikia fusiforme* reduced inflammation, which was not accompanied by a reduction of adipose tissue mass, and improved insulin resistance.

More recently, in the study carried out by Seo et al. [16], which has been previously mentioned in this article, the authors also performed an *in vivo* study with the extract obtained from the macroalgae *Ishige okamurae*. For this purpose, male ICR mice were fed either a standard diet (18% of energy from fat) or a high-fat diet (60% of energy from fat) for 6 weeks. The high-fat diet was supplemented or not with the extract at a dosage of 100 and 300 mg/kg/day. Supplementation of the high-fat diet with the extract led to a significant reduction in body weight gain although without differences between the two doses of the extract. Interestingly, either subcutaneous or visceral fat depots were reduced by the treatment, again independently of the amount of extract offered to the animals. In addition, improvements in fasting blood glucose levels during the 6 weeks of treatment, as well as in the plasma lipid profile at the end of the experimental period were observed when the extract was added to the diet. Thus, decreases in serum TG, free fatty acids (FFA), total cholesterol and the LDL-cholesterol fraction, as well as an increase in the HDL-cholesterol fraction were detected, completely reversing the effect of the high-fat diet feeding.

Supplementation with the extract obtained from *Ishige okamurae* reduced lipid droplet size and lowered the expression of the lipogenic transcription factors C/EBPα and PPARγ in the white adipose tissue of mice fed the high-fat diet, contributing to the lower adiposity observed in the treated groups. Further, the lipolytic pathway was also explored and increased phosphorylations of AMPK and HSL proteins, along with a higher protein expression of ATGL were found. In the case of HSL, the increase on its phosphorylation was only statistically different when the extract was added at the highest concentration (300 mg/kg/day). Concerning the oxidative pathway, supplementation with the extract (300 mg/kg/day) induced increased CPT1 protein expression in the white adipose tissue. All these changes led to a reduction in fat storage in mice fed the high-fat diet supplemented with the extract.

These results show that *Ishige okamurae* is able to reduce body fat accumulation by reducing adipogenesis and by increasing lipolysis and fatty acid oxidation in white adipose tissue.

The same authors performed another study with *Ishige okamurae* extract using a model of obesity-induced type 2 diabetes, as it is characterized not only by hyperglycemia but by a very high fat mass too [24]. In this study, 5-week-old C57B1KsJ-*db*/*db* mice received the vehicle, metformin (140 mg/kg/d) or *Ishige okamurae* extract at doses of 100 or 300 mg/kg/d by oral administration for five weeks, while db/+ mice served as a lean control. Body weight gain was prevented when mice were treated with the high dose of the seaweed extract, as a result of a significant reduction in visceral and subcutaneous adipose tissue depots. Furthermore, the histological evaluation of white adipose tissue showed that cells from control *db*/*db* mice had larger fat droplets than those from metformin and *Ishige okamurae* extract treated groups. Regarding serum parameters, fasting blood glucose concentrations, as well as postprandial blood glucose concentrations, remained lower during the whole period of treatment compared to the control group. Control *db*/*db* mice also showed higher serum HbA1c, total cholesterol and TG concentrations, while these concentrations were lowered in the groups receiving the seaweed supplementation.

In addition, *Ishige okamurae* extract regulated the insulin signaling pathway in white adipose tissue, as mice in both seaweed extract treated groups had higher protein expression and phosphorylation levels of insulin receptor substrate 1 (IRS1), phosphatidylinositol 3-kinase (PI3K) and protein kinase B (Akt). In line with this data, *db*/*db* control group showed lower GLUT4 protein expression, while treatment with 300 mg/kg/d of *Ishige okamurae* extract increased the expression of this glucose transporter in white adipose tissue, probably, as a consequence of the phosphorylation of the intermediates mentioned before. The authors also analyzed the expression of fibroblast growth factor 21 (FGF21), another important metabolic regulator. While *db*/*db* mice showed lower FGF21 protein levels in comparison to db/+ mice, this reduction was less pronounced after *Ishige okamurae* extract administration. Regarding circulating serum FGF21, its concentration was higher in *db*/*db* control group as a result of FGF21 resistance in obesity and diabetes. In the case of the animals receiving the seaweed extract, lower FGF21 levels were found at both doses, reaching values similar to those of the lean control animals. Finally, PGC1α, PPARα and UCP1 protein expression was found to be up-regulated only in mice receiving the high dose of the seaweed extract. These results suggest that *Ishige okamurae* extract can reduce body fat mass, as well as regulate insulin signaling and mitochondrial oxidation and energy expenditure in white adipose tissue.

A great number of studies have used rats, instead of mice, as animal model to test the anti-obesity effects of the macroalgae. In the study reported by Matanjun et al. [25], 10-week-old Sprague-Dawley rats were distributed in four experimental groups: a group fed a normal diet, a group fed a normal diet supplemented with 5% of *Sargassum polycystum*, a group fed a high-cholesterol/high-fat diet (HCF) and a group fed an HCF supplemented with 5% of the same seaweed. Animals were sacrificed after 16 weeks.

At the end of the experimental period whereas rats fed the normal diet did not show differences in body weight gain or adipose tissue due to the inclusion of the seaweed in the diet, rats fed HCF supplemented with *Sargassum polycystum* showed lower body weight gain and lower amount of adipose tissue than non-supplemented HCF fed rats. These results show that seaweeds are effective preventing body weight gain under feeding conditions that promote body fat accumulation, but not under normal feeding. No mechanism justifying these effects were provided.

Plasma total cholesterol and LDL-cholesterol levels were increased in HCF group. In turn, the addition of *Sargassum polycystum* to this diet significantly improved cholesterol parameters, while TG levels remained unchanged. In the case of the animals fed the normal diet, alone or with seaweeds, no differences were observed in these parameters. Based in the results obtained, the authors concluded that seaweed can exert cardioprotective effects when supplemented in the frame of an HCF diet.

González-Torres et al. [26] aimed to study the effect of *Himanthalia elongate*, also called spaghetti seaweed, in restructured pork (RP) meat on adipose tissue of rats fed a diet supplemented with cholesterol and cholic acid. In this study, forty male Wistar rats were split up in four experimental groups: the control group (C, received a normal diet with 15% of RP meat), the sea spaghetti seaweed group (SS, received normal diet with RP meat and mixed with 15% of seaweed), the cholesterol control diet (Ch, received the same diet than the C group, with the addition of 24.34 g/kg cholesterol and 4.9 g/kg cholic acid), and the cholesterol sea spaghetti group (ChSS, received the same diet than the SS group enriched with cholesterol and cholic acid). The rats were maintained under these experimental conditions for 5 weeks.

At the end of the experimental period, dietary inclusion of seaweed (SS group) significantly reduced body weight. This effect was associated with a reduction in food intake, which could be due to the high content in soluble fiber of spaghetti seaweed. The rats fed the diet rich in cholesterol (Ch group) presented lower adipose tissue weight than the C group and, as expected, increased plasma cholesterol levels. Interestingly, the addition of the algae to the diet avoided the reduction in adipose tissue weight and improved hypercholesterolemia.

As far as gene expression is concerned, in adipose tissue (a mix of perirenal and epididymal fat depots) neither Ch nor ChSS diets modified *Lpl* mRNA levels. In addition, cholesterol and cholic acid diet significantly increased *Fas* mRNA levels in the same tissue, though this effect was prevented by the seaweed addition in the cholesterol-supplemented diet. However, regarding Acetyl-CoA carboxylase (*Acc*) gene expression, a significant increase was only appreciated in the ChSS group. Furthermore, cholesterol diet induced *Hsl* mRNA upregulation, which was partially prevented by the cholesterol diet mixed with the seaweed. Interestingly, SS group also showed a decreased *Hsl* gene expression compared with the control group. The authors reported that the *Himanthalia elongate* seaweed mixed with RP meat in the framework of a cholesterol-enriched diet reduced cholesterol levels and regulated the lypolitic/lipogenic adipocyte pathways affected by the cholesterol feeding.

Terpend et al. [27] carried out a study in female Sprague-Dawley rats distributed in four experimental groups: ND group (fed a normal diet), HFD group (fed a high-fat diet) and two additional groups fed the same high-fat diet and supplemented by gavage with 40 mg/kg/day or 400 mg/kg/d of ID-alG™, a seaweed extract obtained from *Ascophyllum nodosum* in which grape extract is used as carrier. The experimental period length was of 8 weeks.

The body weight increase produced by the high-fat diet was diminished by ID-alG™. Moreover, in the case of the higher dose (400 mg/kg/d), the body weight of rats treated with this amount of ID-alG™ reached values of the ND group. Moreover, ID-alG™ consumption decreased the body weight gain in a dose-dependent manner. Concerning fat mass, also a dose-dependent decrease was observed in the treated groups. In serum, TG levels were measured and only the highest dose of ID-alG™ was able to reduce them. No determinations in rat tissues were performed to analyze the mechanisms underlying these effects.

Jang et al. carried out a study using male Sprague-Dawley rats during 6 weeks. Rats were distributed into five experimental groups: a group fed a normal diet, a group fed a high-fat diet and three groups fed the same high-fat diet supplemented with 100, 200 or 400 mg/kg of *Laminaria japonica* Areshoung (LE) [28]. The body weight increase produced by the high-fat diet was reduced by LE in a dose-dependent manner. Moreover, the highest LE dose was able to reduce significantly the body weight gain, when compared to the high-fat group. Concerning different adipose tissue depots, the increase produced by the high-fat diet was suppressed by LE administration. In the histological analysis of epididymal adipose tissue, smaller adipocytes were observed in LE-treated groups when compared to the high-fat diet fed group. Regarding serum parameters, the increase produced by the high-fat diet in TG, total cholesterol, LDL-cholesterol, FFA, TNF-α, leptin, glucose and insulin were attenuated by the administration of LE, reaching values near normal levels. In the case of HDL-cholesterol and adiponectin, LE administration also improved their levels.

In order to better understand the mechanisms underlying these changes, the expression of genes involved in obesity and lipid metabolism was analyzed in adipose tissue. In the case of lipogenesis-related genes, the 200 mg/kg LE dose induced a reduction in the mRNA expression of *Srebp1c*, *Fas*, *Acc1 and Scd-1*. In epididymal adipose tissue, LE supplementation down-regulated the gene expression of *Pparγ* and *Lpl*, while an increase in lipolysis-related genes (*Atgl* and *Hsl*) was observed in the LE treated groups. These results suggest that the anti-obesity effect of LE was due to the increase in fatty acid oxidation accompanied by a decrease in lipogenesis activity.

Grasa-López et al. analyzed the effects of the same macroalgae on lipid metabolism, inflammation and biomarkers of cardiovascular function in 6-week-old male Wistar rats [29]. The animals were divided into 3 experimental groups: the ND group fed in a standard diet (6.2% of energy from fat), the HFD group fed in a high-fat diet (21.2% of energy from fat) and the UP group fed in the same high-fat diet supplemented with 400 mg/kg body weight/day of *Undaria pinnatifida*. The animals were maintained under these experimental conditions for 8 weeks. The seaweed extract was provided daily by intragastric administration to the animals in the UP group, while the animals in the ND and HFD groups received water using the same way of administration. At the end of the experimental period, the animals in the HFD group showed increased body weights compared with the animals in the ND group. In the case of the animals in the UP group, the body weight increase induced by the high-fat feeding was avoided, and the animals showed similar final body weights to rats fed the standard diet. This same pattern of response was observed in the case of the epididymal and mesenteric adipose depots. By contrast, although the weight of the retroperitoneal adipose depot was also significantly lower in the UP group when compared with the HFD group, it remained higher than that observed in the ND group. When image analysis was performed in retroperitoneal adipose tissue, the increased adipocyte size found in the HFD group was prevented by the seaweed treatment.

As far as serum lipid profile is concerned, TG levels were significantly lower in the UP group in comparison with the HFD group. Indeed, the values observed for this parameter in the UP group were also lower than those found in the ND group. Regarding total cholesterol, this parameter was significantly reduced in the UP group when compared with the HFD group, reaching values similar to those found in the ND group. In the case of HDL-cholesterol, which was reduced by the high-fat feeding, rats in the UP group also showed values similar to those from rats in the ND group.

With regard to cytokine levels, the reduction in adiponectin and the increases in leptin, C reactive protein (CRP) and plasminogen activator inhibitor-1 (PAI-1) induced by the high-fat diet were avoided by the seaweed supplementation. In order to gain more insight into the anti-inflammatory effect induced by the seaweed supplementation, the gene expression of different inflammatory markers was measured in the retroperitoneal adipose tissue. The decreased gene expression of adiponectin found in the HFD group was avoided in the UP group, showing values even higher than those reported in the ND group. In the case of leptin and *Il6*, gene expression was significantly increased in the HFD group, while *Undaria pinnatifida* partially avoided this effect.

The expression of several genes involved on lipid metabolism and oxidation was also measured in retroperitoneal adipose tissues. With regard to genes involved in lipid metabolism, increased expression of *Pparγ* was found in the HFD group when compared with the ND group. As far as the UP group is concerned, the expression of this gene was even greater than that found in the HFD group. By contrast, when the gene expression of *Acc* was analyzed, the increased expression observed in the HFD group was prevented by the seaweed supplementation. Indeed, the gene expression found in the UP group was significantly lower than that of the ND group. Finally, when the gene expression of *Ucp1* was studied in retroperitoneal adipose tissue, an increased expression was observed in the animals in the HFD group compared with those in the ND group. In the case of the UP group, the expression was greater than that found in the HFD group.

In view of these results, it can be proposed that *Undaria pinnatifida* supplementation was effective in reducing body and adipose tissue weights, at least in part, as a consequence of decreasing de novo lipogenesis. Moreover, the induction of UCP1 expression in white adipose tissue suggests the activation of browning process. Inflammatory parameters show that seaweed treatment also reduced adipose tissue inflammation, a common feature in obesity. Finally, this macroalgae improved the altered lipid profile induced by the high-fat feeding.

More recently, Kim et al. [30] conducted a study aimed to analyzing the effects of the brown macroalgae *Laminaria japonica* in body weight, serum TG, cholesterol and immunoglobulin G (IgG) levels, and the composition of intestinal microbiota. Interestingly, the authors wanted to elucidate whether different technological treatments could affect the effects induced by the seaweed. Bearing this in mind, 6-week-old male Sprague Dawley rats were fed a diet providing 51% of calories from sucrose and divided in four experimental groups: the control group (received only the diet), the DLJ group (received the diet supplemented with 10% of dried *Laminaria japonica* powder), the HLJ group (received the diet supplemented with 10% of heat-treated *Laminaria japonica*) and the FHLJ group (received the diet supplemented with 10% of heat-treated *Laminaria japonica* + 0.6% of fructooligosaccharides). The animals were maintained under these experimental conditions for 16 weeks.

At the end of the experimental period, decreased body weights were found in all the treated groups when compared to the control group, with no differences among them. Moreover, this body weight reduction occurred without observing reductions in food intake. As in the case of body weight, significantly lower serum TG levels were also appreciated in all the treated groups when compared to the control group, with no differences among them. By contrast, no differences were found in the HDL-cholesterol or in the LDL-cholesterol serum levels. With regard to serum IgG level, this parameter was increased in the HLJ and the FHLJ groups in comparison with the control group.

When intestinal microbiota was analyzed, decreased *Firmicutes* and increased *Bacteroidetes* were observed in all the treated groups when compared with the control group. Among the treated groups, the greatest differences were appreciated in the DLJ group. Moreover, at the genus level, obesity-associated genera (including *Allobaculum*, *Turicibacter* and *Oscillibater*) were significantly decreased in all the treated groups, being the decreases found in the HLJ and FHLJ groups greater than that found in the DLJ group. As far as leanness-associated genera (including *Alistipes*, *Bacteroides* and *Prevotella*) is concerned, significant increases were found in all the treated groups when compared with the control group. With regard to the genera with pathogenic potential (including *Bacteroides*, *Clostridium*, *Escherichia*, *Mollicute* and *Prevotella*), the relative abundances found in all the treated groups were lower than those observed in the control group. In the case of the lactic acid bacterial genera, a significant increase in this functional group was appreciated in the DLJ group when compared with the control group. Finally, greater total butyric acid producing genera were found in the FHLJ group when compared to the control group.

Based on the reported results, the authors concluded that *Laminaria japonica* was effective in reducing the body weight of the rats, and that this effect could be mediated by the modulation of immune response (as shown by the increased serum IgG levels) along with the reductions of the *Firmicutes*/*Bacteroidetes* ratio and the genera with pathogenic potential. Moreover, the increases found in the leanness-associated genera and the lactic acid bacterial were also suggested to participate in the reported body-lowering effect.

Summing-up, the published studies focused on brown algae show that *Ecklonia cava*, *Undaria pinnafitida*, *Ishige okamurae*, *Sargassum polycystum*, *Himanthalia elongate*, *Ascopjyllum nodosum* and *Laminaria japonica* are able to reduce the body fat increase induced by high-fat diets, thus showing a preventive role on obesity. In general terms, the reasons that explain these beneficial effects are the reduction in de novo lipogenesis and the increase in lipid mobilization in adipose tissue. In addition, other mechanisms reported in several studies, such as enhanced skeletal muscle fatty acid oxidation, white adipose tissue browning and a shift towards healthier microbiota profile are also proposed.

In very few cases, controversial results seem to be published. For instance, whereas in the study reported by Park et al. [20] the brown seaweed Jeju *Ecklonia cava* did not induce a reduction in body fat in male C7BL mice, Eo et al. [21] found a significant decrease in adipose tissue weights from different anatomical locations in the same animal model. This fact can be due to differences in several aspects of the experimental design, such as the used seaweed extract dose (200 mg/kg body weight/d in the study reported by Park et al. and 500 mg/kg body weight/d in the study reported by Eo et al.) as well as the experimental period length (11 weeks in the study reported by Park et al. and 22 weeks in the study reported by Eo et al.)

### 3.2. Red Seaweed

Kang et al. [13] analyzed the effect of a red seaweed, *Plocamium telfairiae* extract, on mice fed a high-fat diet. For this purpose, 5-week-old male C57BL/6 mice were fed a normal diet, a high-fat diet or a high-fat diet supplemented with 100 mg/kg body weight/day of *Plocamium telfairiae*. The supplementation with the seaweed led to a lower body weight gain and a decrease in white adipose tissue amount, as well as a reduction in plasma glucose and TG levels. The authors did not assess the mechanisms underlying these effects on adipose tissue.

In another study, the same group aimed to analyze the anti-obesity effect of a *Gelidium amansii* extract in a model of dietary-induced obesity mice fed a high-fat diet [14]. For this purpose, 6-week-old male C57BL/6 mice were fed a normal diet, a high-fat diet, a high-fat diet supplemented with 1% *Gelidium amansii* extract or a high-fat diet supplemented with 3% *Gelidium amansii* extract for 12 weeks. In spite of the observed food and water intake increase, the addition of 3% *Gelidium amansii* extract to the high-fat diet reduced the body and epididymal adipose tissue weights of mice after 10 weeks of supplementation. Regarding plasma biochemical parameters, supplementation with the highest dose of the extract reduced total cholesterol and TG levels. Finally, the histopathological analysis showed that adipocyte size was reduced in the adipose tissue of mice receiving both doses of *Gelidium amansii* extract. To confirm whether the reduction in lipid accumulation and adipocyte size exerted by the macroalgae was mediated by adipogenesis inhibition, the authors tested this seaweed in 3T3-L1 adipocytes, as it has been described in the *in vitro* section of this review article.

Moreover, the same group [31] carried out another study to test the anti-obesity effect of the same algae extract (*Gelidium amansii*) in mice fed a high-fat diet for a shorter period of time. For this purpose, 5-week-old male C57BL/6 mice were fed either a standard diet or a high-fat diet (60% of energy from fat) for 5 weeks in order to induce obesity. Then, the animals fed the standard diet were kept under the same dietary pattern for 8 additional weeks. In the case of the animals fed the high-fat diet, these were divided into 4 experimental groups: a group fed the same high-fat diet and 3 groups fed the same high-fat diet supplemented with different doses of *Gelidium amansii* extract (0.5%, 1% or 2% in the diet) for 8 weeks. At the end of the experimental period, the body weights of the animals receiving the seaweed extract were significantly lower than those found in the non-treated high-fat diet fed animals. Indeed, the body weights of the animals receiving the 1% and 2% doses of the extract were also lower than that observed in the group treated with the lower dose. Apparently, this body weight reduction was not due to decreased body fat since no differences were appreciated among the high-fat diet fed mice (treated and non-treated) in the weights of mesenteric and epididymal adipose depots.

With regard to serum lipids, TG levels were significantly decreased in the treated animals when compared to the non-treated high-fat diet fed mice. Among the treated groups, the reduction in serum TG observed in the group receiving the 2% dose was also significantly greater than that found in the group receiving the lowest dose (0.5%). As far as total cholesterol levels is concerned, significant reductions were only appreciated in the groups receiving the 1% and the 2% doses of the seaweed extract (in a dose-dependent manner). In the case of HDL-cholesterol fraction, higher levels were found in the three treated groups when compared with the non-treated animals. Among the treated groups, the increases found in the groups receiving the 1% and 2% seaweed extract doses were also greater than that observed in the groups receiving the lowest dose. Regarding LDL-cholesterol fraction, the only decreases were appreciated in the groups treated with the 1% and 2% seaweed extract doses, with no differences between them. Similar reductions were also appreciated with regard to serum FFA levels, with lower values in the groups receiving the 1% and 2% seaweed extract doses. As far as adipokine levels are concerned, lower serum leptin levels were found in the three treated groups, being the reduction significantly greater in the groups receiving the highest doses of the seaweed extract (1% and 2%). In the case of serum adipokine, its levels were only increased in the groups receiving the 1% and 2% doses of the seaweed extract, while remained unchanged in the group receiving the lower dose.

In order to gain a better understanding of the mechanisms underlying the aforementioned effects, the protein expressions of different enzymes and transcription factors involved in lipid metabolism were studied in epididymal adipose depot. The results revealed that the protein expressions of SREBP1-c, PPARγ and C/EBPα were significantly decreased by the seaweed extract compared with the non-treated animals, and that the reductions occurred in a dose-dependent manner.

Altogether, the authors conclude that *Gelidium amansii* extract ameliorates obesity and related serum lipid alterations in high-fat diet fed obese mice, and that this effect mainly occurs by the inhibition exerted by the seaweed extract in adipogenesis.

Later on, Park et al. [32] tested the effect of the same macroalgae but in a model of genetic obesity. For this purpose, the authors distributed 5-week-old C57BL/6J *ob*/*ob* obese mice into three experimental groups fed a standard diet for 4 weeks: lean control group (lean C57BL/6J mice), obese control group (*ob*/*ob* C57BL/6J mice) and *ob*/*ob* C57BL/6J mice treated with 0.5% *Gelidium amansii* extract). Mice supplemented with *Gelidium amansii* extract showed lower body weight gain and food intake, suggesting an anti-obesity effect of the macroalgae. When comparing with the obese control group, epididymal and mesenteric adipose tissue weights were significantly reduced after *Gelidium amansii* supplementation, but without reaching the values observed in lean control animals. Plasma lipid levels were also analyzed; after *Gelidium amansii* supplementation, plasma TG and LDL-cholesterol levels were reduced while those of HDL-cholesterol were increased.

In order to analyze the involved mechanisms of action, protein expression of HSL, pAMPK, PPARγ and C/EBPα was assessed in epididymal adipose tissue. The macroalgae supplementation significantly increased the expression of HSL and pAMPK. In addition, *Gelidium amansii* supplementation significantly reduced the adipogenic transcription factors PPARγ and C/EBPα. Based on these results, the anti-obesity effect of this seaweed seems to be due to reduced adipogenesis and increased lipid mobilization.

Choi et al. [33] analyzed the effect of *Gelidium elegans* on adipose tissue of high-fat diet-induced obese mice. For this purpose, male ICR mice (5-week-old) were split up in four experimental groups: the chow diet group (control), the HFD group (60% of energy from fat), the *Gelidium elegans* extract group receiving the low dose (GENS50, received a *Gelidium elegans* extract at a dose of 50 mg/kg/day) and the *Gelidium elegans* extract group receiving the high dose (GENS200, received a *Gelidium elegans* extract at a dose of 200 mg/kg/day). The mice were maintained under these experimental conditions for 7 weeks.

*Gelidium elegans* extract, at both concentrations, avoided the body weight gain induced by the high-fat diet, and it prompted a reduction in abdominal and subcutaneous fat amounts in both tested doses. Interestingly, these effects occurred without differences in total food intake among the groups. Moreover, the high dose of the seaweed extract suppressed the increase induced by the high-fat diet in C/EBPα and PPARγ and PR domain-containing protein 16 (PRMD16) protein expressions. In addition, both *Gelidium elegans* extract doses increased PRDM16 and UCP1 protein expression in brown adipose tissue. Regarding biochemical parameters, GENS200 group showed a significant reduction in serum insulin, glucose and TG levels, parameters that were increased by the high-fat diet. In addition, the HDL-cholesterol level was increased in this group.

These results show that *Gelidium elegans* can prevent high-fat feeding induced fat accumulation, mainly by increasing thermogenic capacity. Moreover, alterations in serum biochemical parameters associated to this feeding pattern can also be ameliorated.

Nakayama et al. [34] investigated the anti-obesity effect of the red seaweed *Palmaria mollis* on body weight gain, hyperlipidemia, hepatic steatosis and visceral adiposity using a mice model of diet-induced obesity. To do so, 6-month-old male NSY/HOS mice were divided in three experimental groups and fed a normal diet or a high-fat diet, supplemented or not with the red seaweed *Palmaria mollis* (2.5% *w*/*w*), for 4 weeks.

The administration of *Palmaria mollis* suppressed the body weight increase induced by the high-fat diet in the second week of the experiment but not in the fourth week, without affecting food intake. The seaweed suppressed visceral adipose tissue accumulation when compared with the high-fat diet fed group, but subcutaneous adipose tissue remained unchanged. Moreover, fasting blood glucose tended to be lower in mice treated with the seaweed than high-fat diet fed mice. In order to describe the mechanisms underlying the anti-obesity effect of this red seaweed, the authors analyzed the expression of transcription factors involved in adipogenesis and lipogenesis in visceral adipose tissue. *Pparγ* was significantly down-regulated in mice treated with *Palmaria mollis*, while no changes were observed in *C/ebpβ* and *C/ebpα* gene expression. In addition, the expression of *Srebp1*, a *Pparγ* downstream gene, was slightly down-regulated. According to the results obtained, the anti-obesity effect of this seaweed may likely be mediated by adipogenesis and lipogenesis inhibition.

*Kappaphycus alvarezii*, widely cultivated for carrageenan production was analyzed by Chin et al. [35] in a study devoted to investigating its anti-obesity mechanisms on diet-induced obese C57BL/6J mice. For this purpose, dried *Kappaphycus alvarezii* powder was added 5% (*w*/*w*) to a high-fat diet (45% of energy from fat) for 6 weeks as a dietary intervention after 10 weeks of high-fat feeding. Therefore, the complete experiment lasted for 16 weeks.

At the end of the experimental period, no statistical differences were observed on body weight gain, total body fat and adipocyte size between animals fed the high-fat diet and those receiving the diet supplemented with *Kappaphycus alvarezii* powder. Further, dietary intervention reduced serum cholesterol concentrations, associated either to a non-statistically significant increase in HDL-cholesterol and/or to a non-significant decrease in LDL-cholesterol fraction. Another effect was a reduction in plasma levels of leptin.

The authors concluded that *Kappaphycus alvarezii* has anti-obesity potential, mainly facilitated by its major soluble fiber, carrageenan and other compounds, which are more effective against the obesity-associated alterations than the whole algae.

The formation of short-chain fatty acid (SCFA) is the result of a complex interplay between diet and gut microbiota [36]. In this regard, obesity is frequently linked to changes in microbiota composition and a reduction in SCFA production [37]. In the study by Chin et al. (2019), high-fat diet-induced changes in SCFA production were partially restored to that of standard diet-fed animals when mice were supplemented with *Kappaphycus alvarezii* powder. Regarding single fatty acids, feeding animals with powder obtained from the whole algae led to an increase in propanoate and a decrease in valerate and isobutyrate. Unfortunately, the authors did not include the group fed with powder obtained from the whole algae in their microbiota analysis and, therefore, no relationship can be established between changes in microbiota and SCFA production [35].

Lee et al. [17] studied the anti-obesity effects of the 60% ethanol extract of the red seaweed *Grateloupia elliptica* in mice fed a high-fat diet. For this purpose, 5–6-week-old male C57 BL/6J mice were divided into four experimental groups: the CD group was fed a chow diet, the HFD group was fed a high-fat diet, the L-GEE group was fed the same high-fat diet supplemented with a low dose of *G. elliptica* (125 mg/kg body weight/day), and the H-GEE group was fed the same high-fat diet supplemented with a high dose of *G. elliptica* (250 mg/kg body weight/day). The treatment was administered orally for 7 weeks.

At the end of the experimental period, L-GEE and H-GEE groups showed a significant reduction in body weight gain when compared with the HFD group. However, white adipose tissue weight increase was only reduced by the highest dose, and this effect was accompanied by a reduction in adipocyte size. Regarding serum parameters, significantly lower levels of TG, total cholesterol and leptin were found in GEE administered animals compared with those in the HFD group. By contrast, no changes in serum insulin levels were found in animals receiving the extract.

To investigate the mechanisms responsible for these effects, the authors analyzed the expression of several adipogenic genes and proteins in white adipose tissue. *Pparγ* gene expression was down-regulated in both treated groups, while a reduction in *c/ebpα* gene expression was only observed in H-GEE group. Moreover, SREBP-1 and PPARγ protein expression was significantly reduced after the oral administration of GEE when compared with the HFD group. FGF21 is a metabolic regulator protein related to lipid and glucose metabolism and energy homeostasis, and its expression was effectively increased by H-GEE treatment. In line with this, the authors also studied the expression of the thermogenic genes *Ucp1* and *Ucp3* in brown adipose tissue, which resulted in a significant increase in mice supplemented with the highest dose of the seaweed extract, suggesting that energy consumption was increased.

These results suggest that the observed reduction in white adipose tissue weight was due, at least in part, to a reduction in adipogenesis in this tissue, as well as due to an increase in thermogenic capacity of brown adipose tissue.

Other authors have used the rat as animal model. Thus, Matanjun et al. [25] analyzed the effect of *Kappaphycus alvarezii* in Sprague-Dawley rats. The rats were distributed in four experimental groups: normal diet group, normal diet group supplemented with 5% of *Kappaphycus alvarezii*, high-cholesterol/high-fat diet group and high-cholesterol/high-fat diet group supplemented with 5% of the same seaweed during 16 weeks.

At the end of the experimental period the animals fed the normal diet (supplemented or not with seaweeds) did not show differences in body weight gain. By contrast, in the animals fed the high-cholesterol/high-fat diet supplemented with seaweed extracts, lower body weight was observed compared with the high-cholesterol/high-fat diet fed non-treated group. The size of adipose tissues was lower in the rats supplemented with the seaweed. Plasma TG, total cholesterol and LDL-cholesterol levels were increased in the high-cholesterol/high-fat diet fed group, while these increases were lowered in the rats supplemented with the seaweed extract. In addition, the HDL-cholesterol levels observed on these animals were similar to those found in rats fed the normal diet. No further analyses were carried out in this study to determine the mechanism of action underlying the effects induced by macroalgae supplementation.

Wanyonyi et al. [38] studied the potential preventive effects of the red macroalgae *Kappaphycus alvarezii* on the metabolic syndrome when used as a dietary supplement. For this purpose, male Wistar rats were divided into three experimental groups and fed a control diet (C) or a high-carbohydrate high-fat diet (H), alone or supplemented with 5% (*w*/*w*) dried and ground *Kappaphycus alvarezii* (HR) for 8 weeks. Rats fed the high-carbohydrate high-fat diet showed symptoms of metabolic syndrome featuring increased body weight, total fat mass, systolic blood pressure, plasma TG and plasma FFA, along with fatty liver. Rats fed the same diet supplemented with the red macroalgae showed normalized body weight and adiposity, lower systolic blood pressure and lower plasma lipids. The authors suggested that these changes were mediated by decreased carbohydrate and lipid absorption, due to gastrointestinal viscosity observed in these rats as a consequence of the algae intake. On the other hand, these rats showed a modulation in the balance of gut *Firmicutes* and *Bacteroidetes*. The ileum of H rats showed infiltration of inflammatory cells, while both C and HR groups showed no damage in the intestine. Moreover, H rats had a lower abundance of *Bacteroidetes* and higher *Firmicutes* compared with C and HR rats. There were no significant differences in the abundance of *Firmicutes* and *Bacteroidetes* between C and HR rats. Therefore, this study demonstrates that *Kappaphycus alvarezii* could effectively reverse metabolic syndrome by inhibiting obesogenic intestinal bacteria and promoting beneficial intestinal bacteria.

Summing-up, the published studies focused on red seaweed show that *Plocamium telfairiae*, *Gelidium amansii*, *Gelidium elegans*, *Palmaria mollis*, *Kappaphycus alvarezii*, *Grateloupia elliptica* and *Kappaphycus alvarezii* are able to reduce the body fat increase induced by high-fat diets, thus showing a preventive role on obesity. The effect on previously established obesity treatment is less clear because, whereas it was effective in a model of genetic obesity, no body-fat-lowering effect was observed in a model of diet-induced obesity. Nevertheless, in this case it should be pointed out that carrageenan extracted from the algae used in this study showed a significant effectiveness. In general terms, the reasons that explain these beneficial effects are a decrease in gut absorption, the reduction in adipogenesis and the increase in lipid mobilization in white adipose tissue, as well as the increase in brown adipose tissue thermogenesis. In addition, other mechanisms reported in several studies, such as a shift towards healthier microbiota profile are also proposed.

### 3.3. Green Seaweeds

In the study reported by Sharma et al. [15], the authors aimed to analyze whether an extract obtained from the green seaweed *Caulerpa okamurae* could exert beneficial effects on obesity. For this purpose, 6-week-old male C57BL/6J mice were fed either in a low-fat diet (LFD group, 10% of energy from fat) or in a high-fat diet (60% of energy from fat) for 10 weeks. The animals fed the high-fat diet received the diet alone (HFD group) or supplemented with 250 mg/kg body weight of *Caulerpa okamurae*. The extract was administered daily by oral gavage.

A significantly lower body weight was found in the mice supplemented with the *Caulerpa okamurae* group when compared with the animals in the HFD group. Similarly, the weight of epididymal and perirenal adipose depots, as well as that of the total fat depots, were reduced in the animals supplemented with the *Caulerpa okamurae* extract. Moreover, when the plasma levels of TG, total cholesterol and FFA were analyzed, the increased values found in the HFD group (in comparison with the LFD group) were significantly lowered by the *Caulerpa okamurae* supplementation.

To better understand the body fat-lowering effect of the *Caulerpa okamurae* extract, protein expression of transcription factors regulating adipogenesis was studied in the adipose tissue. Protein expressions of PPARγ and C/EBPα that had been elevated by the high-fat feeding (HFD group) were decreased by the algae supplementation. Moreover, significantly lower gene expression of *Srebp1-c*, *Fas*, *Acc* and *Cluster of differentiation 36* (*Cd36*) was found in the *Caulerpa okamurae* treated group when compared with the HFD group.

In view of these results, it can be proposed that *Caulerpa okamurae* is able to prevent obesity by reducing adipogenesis as well as by limiting fatty acid availability for TG synthesis in adipose tissue, due to a reduction in de novo lipogeneis and fatty acid uptake from plasma.

Li et al. [39] investigated the effect of the edible siphonaxantin rich green macroalgae *Codium cylindricum* on high-fat diet-induced obesity in male C57BL/6J mice. Animals were distributed in four experimental groups and fed either a low-fat diet (LF, 14% of energy from fat) or a high-fat diet (HFD, 60% of energy from fat), supplemented or not with 1% or 5% of frozen-dried green macroalgae powder for 11 weeks. Supplementation of high-fat diet fed mice with green macroalgae powder resulted in a decrease in body weight gain, but only in those animals receiving the high dose of the extract. This effect was observed in the last week of the experimental period. Indeed, only a decrease in perirenal adipose tissue weight was detected in both treated groups (1% and 5%) compared with the HF diet fed animals, with no changes in none of the other adipose depots nor total white adipose tissue weight.

The authors went through the mechanisms involved in the effects of the supplementation with the green macroalgae. In this regard, they observed a reduction in mRNA levels of genes involved in the lipogenic pathway, *Srebp1*, *Pparγ*, *Fas*, *Stearoyl-CoA desaturase-1* (*Scd1*) and *glucose-6-p dehydrogenase* (*G6pdh*) in these animals compared with the non-supplemented high-fat diet fed mice. Interestingly, the expression of the previously mentioned genes in perirenal adipose tissue tended to be higher in the group supplemented with the extract at 5%, reaching statistical significance in the case of *Pparγ*. Further, when genes involved in the lipid oxidative process were studied, the authors observed a reduction in *Acox1* expression, without changes in that of *Cpt1a* in mesenteric adipose tissue, while in perirenal adipose tissue, an increase in mRNA levels were observed in both genes in the 5% supplemented group. The authors reported that the non-uniform effects on the lipogenic pathways in different white adipose tissue locations implied different anti-obesity mechanisms. On the other hand, an increase in fecal excretion of lipids was found after supplementation with *Codium cylindricum*, due to the high content in fiber. This fact led to increased fecal TG amounts, associated with a decrease in dietary lipid absorption and, consequently, a reduction in white adipose tissue.

In the previously explained study reported by Matanjun et al. [25] *Caulerpa lentillifera* supplementation decreased body weight gain in rats fed an obesogenic diet. Animals fed the high-cholesterol high-fat diet supplemented with the seaweed showed lower amount of adipose tissue than high-cholesterol high-fat diet alone. With regard to serum parameters, the increased LDL-cholesterol level observed in the HCF group was ameliorated by seaweed supplementation, but without reaching the values appreciated in the ND group. In addition, HDL-cholesterol level was increased while that of TG reduced, reaching in these two cases values similar to those found in the normal diet- fed group. Based on these results, the authors concluded that *Caulerpa lentillifera* was cardioprotective and that it was able to reduce body weight gain in rats fed an HCF diet.

Finally, Kumar and co-workers aimed to analyze the effect of two green seaweeds, *Ulva ohnoi* and *Derbesia tenuissima* in a rat model of metabolic syndrome [40]. For this purpose, 9–10-week-old male Wistar rats were fed a high-carbohydrate high-fat diet. Rats previously fed this diet were distributed into six experimental groups: rats fed a standard diet (C group), rats fed a standard diet supplemented with 5% of *Ulva ohnoi* (CUO group), rats fed a standard diet supplemented with 5% of *Derbesia tenuissima* (CDT group), rats fed a high-carbohydrate high-fat diet (H group), rats fed a high-carbohydrate high-fat diet supplemented with 5% of *Ulva ohnoi* (HUO group) and rats fed a high-carbohydrate high-fat diet supplemented with 5% of *Derbesia tenuissima* (HDT group). The experimental period length was of 8 weeks.

The first observation was that food and water intake in the rats of the H group was significantly lower when comparing to the C group, although no statistical differences in body weight gain were found between both groups. Regarding body composition, the authors observed that H rats showed less lean mass and higher total body fat, abdominal fat and visceral adiposity index when compared with the C group. The supplementation with *Ulva ohnoi* reduced total body fat mass, whereas glucose utilization and insulin sensitivity were improved in both HUO and HDT groups, when compared to the H group. Moreover, the high-carbohydrate high-fat diet increased FFA, TG and total cholesterol levels. *Ulva ohnoi* supplementation increased FFA in rats fed this diet while *Derbesia tenuissima* supplementation did not modify FFA values in plasma but reverted the increase in TG and total cholesterol induced by the high-carbohydrate high-fat diet.

Summing-up, the published studies focused on green seaweed show that *Caulerpa okamurae*, *Codium cylindricum*, *Caulerpa lentillifera*, *Ulva ohnoi* and *Derbesia tenuissima* are able to reduce the body fat increase induced by high-fat diets, thus showing a preventive role on obesity. In general terms, the reasons that explain these beneficial effects are a decrease in gut absorption, adipogenesis and lipogenesis, and the increase in fatty acids oxidation in white adipose tissue

## 4. Human Studies

Little information has been reported so far concerning the effects of macroalgae on obesity in humans. The potential of the red edible seaweed *Gelidium elegans* to effectively control body weight and body fat in obese or overweight adults was studied by Kim et al. [41]. For this purpose, 78 volunteers (aged 19-50 years), with a body mass index (BMI) between 23.0 and 30.0 kg/m^2^, were considered as eligible for a double-blind, placebo-controlled, randomized parallel trial, to test the effect of *Gelidium elegans* extract for 12 weeks. Participants in the intervention group (41 subjects) received a daily dose of *Gelidium elegans* extract (1000 mg/day) for 12 weeks, while those in the placebo group (37 subjects) received a placebo in the same regimen as the intervention group. Although the intervention period was 12 weeks, an additional testing session was also included at the 6th week of the intervention to evaluate whether changes occurred earlier than the completion of the whole experimental period. Thus, at baseline, 6th week and 12th week of the experimental period, blood pressure, anthropometric measurement, diet and physical activity assessment, lipid profile, body composition and fat tomography were analyzed.

At the end of the experimental period (week 12), subjects treated with *Gelidium elegans* showed significant decreases in body weight BMI total body fat mass (including visceral abdominal fat), compared to the placebo group. As far as fasting glucose, fasting insulin and TG levels are concerned, no statistical differences were observed between the two groups. However, these parameters were decreased compared with the baseline values after 12 weeks of *Gelidium elegans* intake. In addition, LDL-cholesterol and total cholesterol did not differ between the two groups at any time point. Based on the reported results, the authors suggested that *Gelidium elegans* intake may have a beneficial effect in decreasing body weight, especially fat mass, and TG in overweight and obese subjects.

In this study, the participants were educated to maintain the same level of physical activity during the study as that at baseline, and this was verified throughout the study. However, while in the placebo group, physical activity remained similar or decreased, in the group treated with *Gelidium elegans*, it was increased compared to that of baseline. This is a limitation of the study, and since an increase in physical activity is closely related to body weight loss, this difference could have affected the effects observed in body weight. To avoid this bias, the authors made a statistical adjustment for this variable, among others.

## 5. Concluding Remarks

Data reported in the literature and gathered in the present review show that there is scientific evidence supporting the anti-obesity effect of several brown, red and green macroalgae. *In vitro* studies have been carried out in 3T3-L1 cells by using concentrations of seaweed extracts in the range of 6.25 to 100 µg/mL for pre-adipocytes and 1 to 10 µg/mL for mature adipocytes. All the tested doses have been able to reduce TG accumulation, and in the vast majority of cases, a dose-response pattern has been observed. Nevertheless, it is unclear whether these concentrations replicate the concentrations to which adipose tissue is exposed when providing algae extracts to animals. A great consensus exists in all the reported studies concerning the reduction induced by the seaweed extracts in the expression of transcriptional factors controlling adipogenesis.

With regard to preclinical studies conducted in animal models, those have been carried out in both rats and mice. The doses used in these studies are in the range of 0.5% to 15% (*w*/*w*) in the diet or 100 to 500 mg/kg body weight/d, and the treatment period lengths are in the range of 4 to 16 weeks. Based on the results published and considering that for some algae there is only one study published so far, the most effective range of concentration and treatment duration cannot be established. In the vast majority of these studies, the algae extracts have been provided to animals at the same time than an obesogenic diet. In general terms, it can be stated that macroalgae extracts reduce body fat accumulation in adipose tissue from both visceral and subcutaneous locations. Consequently, the results show the ability of macroalgae to totally or partially prevent obesity development associated to this dietary pattern. In addition, other obesity features, such as dyslipidemia, insulin resistance and fatty liver are also prevented. Among the reported studies, only two works have been performed in animals fed a standard diet and treated with macroalgae extracts. Under this dietary pattern, the results are not as clear as in the case of obesogenic feeding since, while in a study a reduction in adipose tissue was reported, no such effects were observed in the other one. Macroalgae, and more specifically the red seaweed *Gelidium amansii,* have also demonstrated to be effective in reducing body fat accumulation and obesity co-morbidities in a mice model of genetic obesity. More studies carried out in animals receiving the algae extracts after developing obesity are needed in order to determine whether these extracts are effective, not only in preventing obesity but also in managing this disease when stablished. A good experimental model for this purpose would be, for instance, to create a model of diet induced obesity (by using an obesogenic diet) and then to switch the animals to a standard diet supplemented with the algae extract.

Although more research is needed, several mechanisms have been proposed to justify the anti-obesity effects of macroalgae in preclinical studies so far. Apparently, a reduction in food intake is not the main mechanism since this effect has only been observed in a reduced number of studies, although a reduction in gut absorption has been reported by several authors. By contrast, the effects on metabolic pathways in target tissues and organs seem to play a more relevant role. Macroalgae can reduce de novo lipogenesis, thus reducing fatty acid availability for TG synthesis in white adipose tissue. Regarding this mechanism, it should be pointed out that this metabolic pathway in human adipose tissue is not as important as in rodent adipose tissue. This should be taken into account when results from animal models are extrapolated to those obtained in human beings. Moreover, the anti-adipogenic effect observed in cultured cells has also been identified in adipose tissue from animals treated with macroalgae extracts. In a context of obesity, it should be clarified whether this is a positive effect that limits adipose tissue growth and therefore obesity development (or increase), or on the contrary, represents a negative effect for the maintenance of an adequate insulin function. In addition, increased fatty acid oxidation and thermogenic capacity can also contribute to the body fat-lowering effect of macroalgae. A shift towards healthier microbiota profile is also proposed.

Finally, more human studies are warranted to check whether the positive effects observed in rodents are also maintained in human beings in order to determine whether macroalgae can indeed represent an effective tool to prevent and/or to manage obesity.

## Figures and Tables

**Table 1:** Effects of seaweeds in 3T3-L1 pre-adipocytes and mature adipocytes.

Authors	Cell Line	Seaweed and Doses	Experimental Design	Effects	Mechanisms
Kim et al., 2015	3T3-L1 pre-adipocytes	*Sargassum thunbergii* (brown seaweed) extract 10, 50 and 100 µg/mL	Cells treated during the 8 days of the differentiation period	↓ Adipogenesis	↓ PPARγ, SREBP1c and C/EBPα gene and protein expressions ↓ RXRα, RXRβ, LXRα, LXRβ protein expression
Kang et al., 2016	3T3-L1 pre-adipocytes	Brown, green and red algae extracts (100 µg/mL) *Plocamium telfairiae* (red seaweed) extract (25, 50 and 100 µg/L)	Cells treated during the 8 days of the differentiation period	↓ Adipogenesis	↓ PPARγ, C/EBPα, SREBP1c, FABP4 protein expression
Kang et al., 2016	3T3-L1 pre-adipocytes	*Gelidium amansii* (red seaweed) extract (50 and 100 µg/mL)	Cells treated during the 8 days of the differentiation period	↓ Adipogenesis	↓ PPARγ, C/EBPα (100 µg/mL) and SREBP1c protein expression
Sharma et al., 2017	3T3-L1 pre-adipocytes	*Caulerpa okamurae* (green seaweed) extract (25, 50, 125, 250 and 500 µg/mL)	Cells treated during the 8 days of the differentiation period	↓ Adipogenesis	↓ PPARγ and C/EBPα protein expression (50 and 250 µg/mL) ↓ SREBP1c protein expression (<250 µg/mL)
Martínez-Villaluenga et al., 2018	3T3-L1 mature adipocytes	*Undaria pinnatifida* (Brown seaweed) extract (1 and 10 µg/mL)	Cells treated on day 8 of differentiation (during 24 h)	↓ TG accumulation	No information is provided
Seo et al., 2018	3T3-L1 pre-adipocytes	*Ishige okamurae* (brown seaweed) extract (6.25, 12.5 and 25 µg/mL)	Cells treated during the 8 days of the differentiation period	↓ Adipogenesis	↓ PPARγ, C/EBPα protein expression ↑ phosphorylated-AMPK, ATGL and phosphorylated-HSL protein expression ↑ CPT1 (25 µg/mL)
Lee et al., 2020	3T3-L1 pre-adipocytes	*Grateloupia elliptica* (red seaweed) extract (25, 50, 100 and 200 µg/mL)	Cells treated during the 8 days of the differentiation period	↓ TG accumulation ↓ Adipogenesis	↓ PPARγ, SREBP1, FABP4 protein expression

AMPK: AMP-activated protein kinase, ATGL: adipose triglyceride lipase, C/EBPα: CCAAT-enhancer-binding protein α, CPT1: carnitine palmitoyltransferase I, FABP4: fatty acid-binding protein 4, HSL: hormone sensitive lipase, LXRα: liver X receptor α, LXRβ: liver X receptor β, PPARγ: peroxisome proliferator activated receptor γ, RXRα: retinoid X receptor α; RXRβ: retinoid X receptor β; SREBP1c: sterol regulatory element-binding protein 1 c, TG: triglycerides. ↓: decreased, ↑: increased.

**Table 2:** *In vivo* effects of brown seaweeds in different animal models.

Authors	Animal Model	Seaweed Dose and Experimental Period Length	Dietary Experimental Groups	Effects	Mechanisms
Okada et al., 2011	Male KK-Ay mice (3-week-old)	*Undaria pinnatifida* lipids 0.2% in water or 1% in diet 4 weeks	Standard diet	↓ Body weight (in water) ↓ Mesenteric AT (in diet)	No changes in UCP1 gene and protein expressions
Park et al., 2012	Male C7BL mice (6-week-old)	Jeju *Ecklonia cava* Gijang *Ecklonia cava* 200 mg/kg BW/day 11 weeks	Standard diet High fat diet High fat diet + Jeju *Ecklonia cava* High fat diet + Gijang *Ecklonia cava*	Jeju *Ecklonia* cava No effects Gijang *Ecklonia cava* ↓ Body weight ↓ % Total fat, ↓ % Epididymal AT ↓ % Mesenteric AT ↓ % Perirenal AT ↓ % Subcutaneous AT ↓ Plasma TC, LDL-c	Gijang *Ecklonia cava* (epididimal AT) *↑ Pparγ2*, *C/ebpα*, *Srebp1c*, *Fas* gene expression *↑* phosphorylated-AMPK/total-AMPK ↓ *Tnf-α*, *Il-1β*, and *F4/80* gene expression
Eo et al., 2015	Male C7BL mice (4-week-old)	Jeju *Ecklonia cava* extract 100 or 500 mg/kg BW/day 22 weeks	Standard diet High fat diet High fat diet + 100 mg/kg BW/day Jeju *Ecklonia cava* (from week 10) High fat diet + 500 mg/kg BW/day Jeju *Ecklonia cava* (from week 10)	↓ Body weight (500 mg/kg BW/day) ↓ Total and retroperitoneal AT (500 mg/kg BW/day) ↓ Sum of visceral and mesenteric AT ↓ Epididymal AT (100 mg/kg BW/day) ↓ Plasma TG, TC ↓ Plasma leptin (100 mg/kg BW/day) ↓ Plasma leptin/adiponectin (100 mg/kg BW/day)	No information is provided
Park et al., 2015	Male C7BL mice (6-week-old)	Gijang *Ecklonia cava* extract 200 mg/kg BW/day 16 weeks	Standard diet High fat diet High fat diet + Gijang *Ecklonia* cava (from week 6)	↓ Body weight ↓ Total fat, visceral and subcutaneous AT weights ↓ Serum TC and LDL-c	No information is provided
Oh et al., 2016	Male C57BL/6 mice (5-week-old) and their bone-marrow-derived immune cells isolated from femoral and tibia bone-marrow	*Undaria pinnafitida* (UP), *Laminaria japonica* (LJ), *Sargassum fulvellum* (SF) and *Hizikia fusiforme* (HF) 5% in the diet 16 weeks Bone-marrow-derived immune cells were isolated from a representative mouse of each experimental group. Cells were differentiated into macrophages (BMDM) and stimulated or not with LPS for 24 h.	Low-fat diet (LFD; 10% of energy from fat) High-fat diet (HFD; 60% of energy from fat) HFD + UP 5% HFD + LJ 5% HFD + SF 5% HFD + HF 5%	↑ Body weight gain (HFD + UP vs. HFD) ↑ Subcutaneous AT weight (HFD + UP vs. HFD) ↓ Plasma adiponectin (HFD + UP) ↑ Plasma leptin (HFD + LJ, HFD + SF and HFD + HF) ↓ Blood glucose (HFD + LJ, HFD + SF and HFD + HF) ↓ IR (HFD + LJ)	↓ CLS number in gonadal adipose tissue ↓ Adipocyte size of gonadal AT ↓ Il-1β (all groups-derived BMDM) ↓ Il-6 (HFD + LJ-derived BMDM)
Seo et al., 2018	Male ICR mice (4-week-old)	*Ishige okamurae* extract (IOE) 100 mg/kg BW/day 300 mg/kg BW/day 6 weeks	Standard diet (SD; 18% of energy as fat) High-fat diet (HFD; 60% of energy as fat) HFD + IOE 100 mg/kg BW/day HFD + IOE 300 mg/kg BW/day	↓ Subcutaneous and visceral WAT mass ↓ Fasting blood glucose ↓ Plasma TG, FFA, TC and LDL-c ↑ HDL-c ↓ Adipocyte size in visceral WAT ↓ Adipogenesis in visceral WAT ↑ Propanoate ↓ Valerate and isobutyrate	↓ Protein expression of C/EBPα and PPARγ (WAT) ↑ phosphorylated-AMPK ↑ ATGL and phosphorylated-HSL protein expressions (300 mg/kg BW/day) ↑ CPT1 protein expression
Seo et al., 2019	Male *db*/*db* and lean db/+ mice (5-week-old)	*Ishige okamurae* extract (IOE) 100 mg/kg BW/day 300 mg/kg BW/day (oral administration) 5 weeks	db/+ (lean control) *db*/*db* control *db*/*db* + Metformin 140 mg/kg BW/day (positive control) *db*/*db* + IOE 100 mg/kg BW/day *db*/*db* + IOE 300 mg/kg BW/day	↓ Body mass gain (300 mg/kg BW/day) ↓ Visceral and subcutaneous WAT masses (300 mg/kg BW/day) ↓ Adipocyte fat droplet size ↓ Fasting and postprandial blood glucose (300 mg/kg BW/day) ↓ Serum HbA1c, TC (300 mg/kg BW/day), TG ↓ Serum FGF21	↑ GLUT4 protein expression in WAT (300 mg/kg BW/day) ↑ Phosphorylated-IRS, phosphorylated-PI3K, phosphorylated-Akt protein expression in WAT ↑ FGF21 protein expression in WAT ↑ PGC1α, UCP1, PPARα protein expression in WAT (300 mg/kg BW/day)
Matanjun et al., 2010	Male Sprague-Dawley rats (10-week-old)	*Sargassum polycystum* (SP) 5% in the diet 16 weeks	Standard diet Standard diet + SP HCF (1 g cholesterol + 24 g fat/100 g diet) HCF + SP	Standard diet No effects HCF diet ↓ Body weight ↓ Weight gain ↓ AT ↓ Plasma TC and LDL-c ↑ Plasma HDL-c	No information is provided
González-Torres et al., 2012	Male Wistar rats (no age provided)	*Himanthalia elongate* 15% in restructured pork (RP) meat 5 weeks	Control diet (C) + 15% RP meat (C group) C + 15% RP + 15% seaweed (SS group) C + cholesterol (24.34 g/kg) and cholic acid (4.9 g/kg) + 15% RP (Ch group) C + cholesterol and cholic acid + 15% RP + 15% seaweed (ChSS group)	↓ Body weight (SS group) ↓ Food intake (SS group) ↓ AT weight (Ch group) ↓ Plasma TC level (ChSS group)	↓ *Fas* gene expression (ChSS group) ↑ *Acc* gene expression (ChSS group) ↓ *Hsl* gene expression (ChSS group)
Terpend et al., 2012	Female Sprague-Dawley rats (170–180 g; no information concerning the age)	ID-alG™, a seaweed extract produced from *Ascopjyllum nodosum* and having grape extract as a carrier 8 weeks	Standard diet HFD group HFD + 40 mg/kg BW/day ID-alG™ HFD + 400 mg/kg BW/day ID-alG™	↓ Body weight ↓ Body weight gain ↓ Body fat mass ↓ Serum TG (high dose)	No information is provided
Jang et al., 2013	Male Sprague-Dawley rats (no information concerning the age)	*Laminaria japonica* Areshoung (LE) 100, 200 and 400 mg/kg 6 weeks	Standard diet High-fat diet (HFD) HFD + 100 mg/kg LE HFD + 200 mg/kg LE HFD + 400 mg/kg LE	↓ Body weight ↓ AT depots ↓ Adipocyte size ↓ TG, TC, LDL-c, FFA, TNF-α, leptin, glucose and insulin serum levels ↑ HDL-c and adiponectin	↓ *Srebp1c*, *Fas*, *Acc1* and *Scd-1* gene expression ↓ *Pparγ* and *Lpl* gene expression ↑ A*tgl* and *Hsl* gene expression
Grasa-López et al., 2016	Male Wistar rat (6-week-old)	*Undaria pinnatifida* 400 mg/kg BW/day 8 weeks	Standard diet (NF) High-fat diet (HFD; 21.2% of energy from fat) High-fat diet + *Undaria pinnatifida* (UP)	↓ Body weight ↓ Epididymal, mesenteric and retroperitoneal AT weights ↓ Adipocyte size ↓ TG and TC serum levels ↑ Serum HDL-c levels ↑ Serum adiponectin levels ↓ Serum leptin, CRP and PAI-1 levels	↑ Adiponectin gene expression (retroperitoneal adipose tissue) ↓ Leptin and *Il-6* gene expression (retroperitoneal adipose tissue) ↑ *Pparγ* gene expression (retroperitoneal adipose tissue) ↓ *Acc* gene expression (retroperitoneal adipose tissue) ↑ *Ucp1* gene expression (retroperitoneal adipose tissue)
Kim et al., 2018	Male Sprague-Dawley rats (6-week-old)	*Laminaria japonica* 10% dried + powdered or heat-treated in the diet 16 weeks	High-carbohydrate diet; 51% of energy from sucrose) + *Laminaria japonica*	↓ Body weight ↓ Serum TG levels ↑ Serum IgG levels	↓ *Firmicutes*/*Bacteroidetes* ratio and obesity-associated genera ↓Genera with pathogenic potential ↑ Leanness-associated genera and LAB genera

ACC: Acetyl-CoA carboxylase, Akt: protein kinase B, AMPK: AMP-activated protein kinase, AT: adipose tissue, ATGL: adipose triglyceride lipase, BMDM: bone-marrow-derived macrophages, BW: body weight, C/EBPα: CCAAT-enhancer-binding protein α, CLS: Crown-like structures, CPT1: carnitine palmitoyltransferase 1, CRP: C reactive protein, F4/80: homologue in mouse to epidermal growth factor-like 1 in humans, FAS: fatty acid synthase, FFA: free fatty acid, FGF21: fibroblast growth factor 21, GLUT4: glucose transporter 4, HbA1c: glycated hemoglobin, HDL-c: high-density lipoprotein cholesterol, HSL: hormone sensitive lipase, ICR: Institute of Cancer Research, IgG: immunoglobulin G, Il-1β: interleukin 1 β, Il-6: interleukin-6, IRS: insulin receptor substrate LAB: lactic acid bacteria, LDL-c: low-density lipoprotein cholesterol, LPL: Lipoprotein lipase, LPS: lipopolysaccharide, PAI-1: plasminogen activator inhibitor-1, PGC1α: Peroxisome proliferator-activated receptor gamma co-activator 1α, PI3K: phosphatidylinositol 3-kinase, PPARα: perosyxome proliferator activated receptor α, PPARγ: perosyxome proliferator activated receptor γ, SCD-1: stearoyl-CoA desaturase-1, SREBP1c: sterol regulatory element-binding protein 1 c, TC: total cholesterol, TG: triglycerides, TNF-α: tumor necrosis factor-α, UCP1: uncoupling protein 1, WAT: white adipose tissue, ↓ decreased, ↑: increased.

**Table 3:** *In vivo* effects of red seaweeds in different animal models.

Authors	Animal Model	Seaweed Dose and Experimental Period Length	Dietary Experimental Groups	Effects	Mechanisms
Kang et al., 2016	Male C57BL/6 mice (5-week-old)	*Plocamium telfairiae* extract (PTE) 100 mg/kg BW/day 9 weeks	Standard diet High-fat diet (HFD) HFD + 100 mg /kg BW/day	↓ Body weight ↓ AT ↓ Serum glucose levels ↓ Serum TG levels	No information is provided
Kang et al., 2016	Male C57BL/6 mice (6-week-old)	*Gelidium amansii* extract 1% and 3% in the diet 12 weeks	Standard diet High-fat diet (HFD; 45% of energy from fat) HFD + 1% seaweed extract HFD + 3% seaweed extract	↓ Body weight ↓ Epididymal AT ↓ Adipocyte size ↓ Serum TG levels ↓ Serum TC levels	No information is provided
Kang et al., 2017	Male C57BL/6 mice (5-week-old)	*Gelidium amansii* extract 0.5%, 1% and 2% in the diet 8 weeks	Standard diet High-fat diet (HFD); 60% of energy from fat) HFD + 0.5% seaweed extract HFD + 1% seaweed extract HFD + 2% seaweed extract	↓ Body weight ↓ Weight gain ↓ Serum TG levels ↓ Serum TC levels (HFD + 1% and HFD + 2% groups) ↑ Serum HDL-c levels ↓ Serum LDL-c levels (HFD + 1% and HFD + 2% groups) ↓ Serum FFA levels (HFD + 1% and HFD + 2% groups) ↓ Serum leptin levels ↑ Serum adiponectin levels (HFD + 1% and HFD + 2% groups)	↓ PPARγ, SREBP1C and C/EBPα protein expressions ↑ HSL protein expression
Choi et al., 2017	Male ICR mice (5-week-old)	*Gelidium elegans* (GENS) 7 weeks	Chow diet (control group) High-fat diet (HFD; 60% of energy from fat) HFD + GENS 50 mg/kg BW/day (GENS50) HFD + GENS 200 mg/kg BW/day (GENS200)	↓ Body weight gain ↓ Subcutaneous and abdominal fat amount ↓ Serum insulin and TG levels (GENS200) ↑ HDL-c level (GENS200)	↓ C/EBPα and PPARγ protein expression (GENS200) ↑ PRDM16 and UCP1 protein expression in BAT
Park et al., 2017	Male lean and *ob/ob* C57BL/6J mice (5-week-old)	*Gelidium amansii* extract (GAE) 0.5% in the diet 4 weeks	Standard diet Lean mice (lean control group) *ob/ob* mice (*ob/ob* control group) *ob/ob* mice + GAE (0.5%)	↓ Body weight gain ↓ Food intake ↓ Food efficiency ratio ↓ Epididymal AT ↓ Mesenteric AT ↓ Serum TG, LDL-c and ↑HDL-c ↑ Serum adiponectin	↑ HSL and phosphorylated-AMPK protein expression in epididymal adipose tissue ↓ PPARγ and C/EBPα protein expression in epididymal adipose tissue
Nakayama et al., 2018	Male NSY/HOS mice (a type 2 diabetes mellitus strain) (6-month-old)	*Palmaria mollis* 2.5% in the diet 4 weeks	Normal diet High-fat diet (60% of energy from fat) High-fat diet + *Palmaria mollis*	↓ Visceral AT ↓ Fasting glucose	↓ *Ppar*γ and *Srebf1* gene expression in visceral adipose tissue
Chin et al., 2019	Male specific-pathogen free C57BL/6J mice (4-week-old)	*Kappaphycus alvarezii* (KA) dry powder 5% 10 weeks to induce obesity followed by 6 weeks of dietary intervention	Low-fat diet (LFD; 10% of energy as fat) High-fat diet (HFD; 45% of energy as fat HFD + 5% KA (KA)	↓ Plasma leptin and adiponectin levels No changes in body fat and adipocyte size	No information is provided
Lee et al., 2020	Male C57BL/6J mice (5-6-week-old)	*Grateloupia elliptica* (GEE) 125 and 250 mg/kg BW/day 7 weeks	Chow diet (CD) High-fat diet (HFD) HFD + 125 mg/kg BW/day GEE (L-GEE) HFD + 250 mg/kg BW/day GEE (H-GEE) HFD + 125 mg/kg BW/day *Garcinia cambogia* (GCE) as positive control	↓ Body weight ↓ WAT weight (H-GEE) ↓ Adipocyte size in WAT (H-GEE) ↓ Serum TG, TC and leptin	↓ *C*/*ebp*α gene expression in WAT (H-GEE) ↓ *Pparγ* gene expression in WAT ↓ SREBP1 and PPARγ protein expression in WAT ↑ FGF21 protein expression in WAT (H-GEE) ↑ *Ucp1* and *Ucp3* gene expression in BAT (H-GEE)
Matanjun et al., 2010	Male Sprague-Dawley rats (10-week-old)	*Kappaphycus alvarezii* 5% in the diet 16 weeks	Standard diet Standard diet +Ka HCF (1 g cholesterol + 24 g fat/100 g diet) HCF + Ka	Standard diet No effects HCF diet ↓ Body weight ↓ Weight gain ↓ AT ↓ Plasma TC and LDL-c ↑ Plasma HDL-c ↓ Plasma TG	No information is provided
Wanyonyi et al., 2017	Male Wistar rats (8–9-week-old)	*Kappaphycus alvarezii* 5% in the diet 8 weeks	Control diet High-fat and high-carbohydrate diet High-fat and high-carbohydrates + *Kappaphycus alvarezii*	↓ Body weight gain, abdominal circumference, retroperitoneal, omental, total visceral and total AT weight ↓ Blood glucose and TG	↓ Absorption ↑ *Bacteroidetes* ↓ *Firmicutes*

AMPK: AMP-activated protein kinase, AT: adipose tissue, BAT: brown adipose tissue, BW: body weight, C/EBPα: CCAAT-enhancer-binding protein α, FFA: free fatty acid, FGF21: fibroblast growth factor-21, HDL-c: high-density lipoprotein cholesterol, HSL: hormone sensitive lipase, LDL-c: low-density lipoprotein cholesterol, PPARγ: perosyxome proliferator activated receptor γ, PRDM16: PR domain-containing16, SREBF1: Sterol Regulatory Element Binding Transcription Factor 1, SREBP1: sterol regulatory element-binding protein 1 c, TC: total cholesterol, TG: triglycerides, UCP1: uncoupling protein 1, UCP3: uncoupling protein 3, WAT: white adipose tissue, ↓ decreased, ↑: increased.

**Table 4:** *In vivo* effects of green seaweeds in different animal models.

Authors	Animal Model	Seaweed Dose and Experimental Period Length	Dietary Experimental Groups	Effects	Mechanisms
Sharma et al., 2017	Male C57BL/6J mice (6-week-old)	*Caulerpa okamurae* 250 mg/kg/BW/day 10 weeks	High-fat diet (HFD; 60% of energy from fat) HFD + *Caulerpa okamurae*	↓ Body weight ↓ Total, epididymal and perirenal AT weights ↓ Plasma TG, TC and FFA levels	↓ PPARγ and C/EBPα protein expression (AT) ↓ *Srebp-c*, *Fas, Acc* and *Cd36* gene expression in AT
Li et al., 2018	Male C57BL/6 mice (4-week-old)	*Codium cylindricum* 1% and 5% dried powder in the diet 11 weeks	High-fat diet (HF; 60% of energy from fat) HF + *Codium cylindricum* 1% (1GA) HF + *Codium cylindricum* 5% (5GA)	↓ Body weight gain (5GA vs. HF; from week 10) ↓ Perirenal WAT ↑ Fecal TG and FFA in 5GA	↓ Lipid absorption ↓ *Srebp1*, *Fas*, *Scd-1*, *G6pdh, Ppar*γ and *Acox1* gene expressions in 5GA (mesenteric WAT) ↓ *Acox1* gene expression in 1GA (mesenteric WAT) ↑ *Ppar*γ and *Cpt1a* gene expressions in 5GA (perirenal WAT) ↑ *Ppargc1a* gene expression in 5GA (mesenteric WAT)
Matanjun et al., 2010	Male Sprague-Dawley rats (10-week-old)	*Caulerpa lentillifera* 5% in the diet 16 weeks	Standard diet Standard diet + Cl HCF (1 g cholesterol+ 24 g fat/100 g diet) HCF + Cl	Standard diet No effects HCF diet ↓ Body weight ↓ Weight gain ↓ AT ↓ Plasma TC and LDL-c ↑ Plasma HDL-c ↓ Plasma TG	No information is provided
Kumar et al., 2015	Male Wistar rats (9–10-week-old)	*Ulva ohnoi* *Derbesia tenuissima* 5% in the diet 8 weeks	Standard diet (C) C + *Ulva ohnoi* (CUO) C + *Derbesia tenuissima* (CDT) HCHF (H) H + *Ulva ohnoi* (HUO) H + *Derbesia tenuissima* (HDT)	↓ Total fat mass (HUO group) ↑ Glucose utilization and insulin sensitivity (HUO and HDT vs. H group) ↑ FFA (HUO group) ↓ TG and TC (HDT vs. H group)	No information is provided

ACC: Acetyl-CoA carboxylase, Acox1: acyl-CoA oxidase1, AT: adipose tissue, BW: body weight, Cd36: cluster of differentiation 36, C/EBPα: CCAAT-enhancer-binding protein α, CPT: carnitine palmitoyltransferase, FAS: fatty acid synthase, FFA: free fatty acid, G6PHD: glucose 6 phosphate dehydrogenase, HDL-c: high-density lipoprotein cholesterol, PPARγ: perosyxome proliferator activated receptor γ, Ppargc1α: PPAR gamma coactivator 1 alpha, SCD-1: stearoyl-CoA desaturase 1, SREBP1: sterol regulatory element-binding protein, TC: total cholesterol, TG: triglycerides, WAT: white adipose tissue, ↓: decreased, ↑: increased.
